# Takayasu arteritis presenting with dysphagia and morning stiffness: a diagnostic challenge in a middle-aged woman

**DOI:** 10.1186/s43044-026-00774-z

**Published:** 2026-09-05

**Authors:** Niloofar Najafi, Seyedeh Tahereh Faezi, Hakimeh Sadeghian, Khashayar Danandeh

**Affiliations:** 1 Rheumatology Research Center, Tehran University of Medical Sciences, Tehran, Islamic Republic of Iran; 2 Department of Echocardiography, Shariati Hospital, Tehran University of Medical Sciences, Tehran, Islamic Republic of Iran

**Keywords:** Takayasu arteritis, Dysphagia, Rheumatology, Morning stiffness, Case report

## Abstract

**Background:**

Takayasu arteritis is a rare, chronic vasculitis affecting large arteries, primarily the aorta and its principal branches. The illness frequently presents with non-specific systemic symptoms, leading to a postponed diagnosis. Dysphagia resulting from aortic involvement is a rare manifestation and may resemble other cardiovascular or gastrointestinal disorders.

**Case presentation:**

We report a 45-year-old Iranian woman with a one-year history of exertional dyspnoea, intermittent dysphagia for solid food, and diffuse musculoskeletal pain with morning stiffness. Initial evaluation showed elevated inflammatory markers, moderate aortic regurgitation, and aneurysmal dilation of the ascending aorta and aortic arch, and infective endocarditis was initially suspected. After serial negative blood cultures and resolution of a suspicious valvular finding on repeat transoesophageal echocardiography, upper gastrointestinal endoscopy and endoscopic ultrasonography localised her dysphagia to extrinsic oesophageal compression by the dilated thoracic aorta. Computed tomography angiography confirmed a non-dissecting aneurysm of the ascending aorta and arch, and whole-body ¹⁸F-FDG PET demonstrated markedly increased metabolic activity along the thoracic aorta and its supra-aortic branches, consistent with active large-vessel vasculitis. After systematic exclusion of infective and other inflammatory mimics, a clinical diagnosis of Takayasu arteritis was made.

**Conclusion:**

This case illustrates an uncommon presentation of Takayasu arteritis with dysphagia produced by extrinsic oesophageal compression from thoracic aortic aneurysmal dilatation. Constitutional symptoms, elevated inflammatory markers, and unexplained cardiovascular findings should prompt evaluation for large-vessel vasculitis. Early multimodal imaging and a systematic approach to excluding infective and other inflammatory mimics are essential for accurate diagnosis and timely treatment.

**Supplementary Information:**

The online version contains supplementary material available at https://doi.org/10.1186/s43044-026-00774-z.

## Introduction

Takayasu’s arteritis (TA) is a rare and chronic condition that involves large vessels. Inflammatory vasculitis is primarily characterised by granulomatous inflammation; subsequently, stenosis, aneurysmal dilation, and occlusion are observed in progressive stages [1]. The aorta and its main branches, especially the renal, carotid, and subclavian arteries, are frequently impacted by this condition [2]. Epidemiological studies show that Asian women of childbearing age, mostly in their second and third decades of life, are thought to be more prone to this illness [3]. However, the female-to-male ratio of TA varies across regions, and men and women have differing clinical presentations, including the distribution of vascular lesions [4]. Although recently several cases have been explored around the world, Japan has the most cases globally [2, 4, 5].

Diagnosis of TA can be difficult owing to its non-specific clinical presentation and the absence of a single confirmatory test. Angiography is regarded as the gold standard for visualising vascular involvement and determining disease severity [6]. It can detect stenotic lesions, aneurysms, and vascular occlusions. Ultrasound and Doppler methods are used to measure artery wall thickness, diagnose flow irregularities, and track disease progression. In unusual locations, such as the vertebral arteries, the combination of PET and MRI imaging could help in the identification of active TA. An appropriate diagnosis is dependent on a combination of clinical factors and imaging techniques. Early diagnosis and treatment are critical to avoiding complications and improving patient outcomes [7].

In this case report study, we introduced a middle-aged Iranian woman with an uncommon initial presentation diagnosed with TA.

## Case presentation

### Initial presentation

A 45-year-old woman of Iranian nationality, with a history of minor beta-thalassaemia trait and mild hypertension and no family history of autoimmune, vasculitic, or cardiovascular disease, presented with a triad of symptoms evolving over approximately one year: exertional dyspnoea with retrosternal chest pain and palpitations (onset ~ 12 months before admission); progressive intermittent dysphagia for solid food, partially relieved by water and without odynophagia, haematemesis, or weight loss (onset ~ 6 months before admission); and diffuse musculoskeletal pain with approximately ten minutes of morning stiffness (onset ~ 8 months before admission). She denied limb or jaw claudication, headache, rash, oral ulcers, and ocular symptoms, and was taking only folic acid and cholecalciferol.

Outpatient transthoracic echocardiography at symptom onset showed moderate aortic insufficiency with preserved left ventricular function (LVEF 55%); aortic diameters were not documented at that time and could not be retrieved on retrospective review, a limitation of this case description. Concurrently elevated ESR/CRP raised concern for infective endocarditis, and empirical antibiotics were started without sustained benefit. Given the lack of response and progression of symptoms, she was hospitalised for comprehensive evaluation.

On admission, blood pressure was 170/86 mmHg and pulse 104 bpm, with an early diastolic murmur consistent with aortic regurgitation; chest X-ray was normal, and joint, muscle, and skin examination were unremarkable. Four-limb blood pressure measurement (Table 1) showed a 10 mmHg inter-arm systolic difference, below the > 10 mmHg threshold used in the 1990 ACR classification criteria and not corroborated by computed tomography angiography (CTA), which later showed patent aortic arch branches; this finding was therefore considered non-specific rather than definite evidence of subclavian involvement.

**Table 1:** Four-limb blood pressure measurements recorded during rheumatological assessment

Site	BP (mmHg)	PR (bpm)	SpO₂ (%)
Left arm	120/80	78	95
Right arm	110/85	78	95
Left ankle	120/80	—	—
Right ankle	110/80	—	—

The 10 mmHg inter-arm systolic discrepancy did not exceed the > 10 mmHg 1990 ACR threshold and was not corroborated by CTA; the ipsilateral (left) arm-ankle pressures were equal rather than showing the physiological ankle-arm gradient, concordant with normal-calibre abdominal aorta and lower-limb arteries on CTA. Both findings were considered non-specific

### Exclusion of infection and other major mimics

Admission laboratory investigations (Table 2) showed normocytic anaemia and active systemic inflammation (ESR 58 mm/h, CRP 24 mg/L), with normal renal and hepatic function and a normal urinalysis. Two independent sets of blood cultures were negative, effectively excluding bacteraemia. An extended immunology and infectious screen, antinuclear and anti-double-stranded DNA antibodies, complement (C3, C4, CH50), antiphospholipid antibody panel, serum ACE, IgG and IgG4, VDRL, interferon-gamma release assay for tuberculosis, and hepatitis B/C and HIV serology, was unremarkable throughout (Table 2, footnote).

Initial transoesophageal echocardiography (TOE) on hospital day 10 showed moderate aortic insufficiency and a 6 mm focal thickening of the non-coronary cusp ‘suspicious for vegetation’, which, together with the elevated inflammatory markers, fulfilled several modified Duke criteria [19] and prompted continued empirical intravenous antibiotics (vancomycin and ceftriaxone) for possible infective endocarditis. After completion of the antibiotic course and confirmation of negative cultures, repeat TOE no longer showed any vegetation, effectively excluding infective endocarditis; moderate aortic insufficiency and a dilated aortic arch (43 mm) persisted.

In parallel, a structured differential diagnosis was constructed for the combination of systemic inflammation, non-dissecting aortic aneurysmal dilatation, aortic regurgitation, and dysphagia. Infectious (mycotic) aortitis, giant cell arteritis, IgG4-related aortitis, Behçet disease, isolated/idiopathic aortitis, and degenerative (‘dysphagia aortica’) aneurysm were each considered and judged less likely than an inflammatory large-vessel process [8–14], principally on the basis of the patient’s age, the diffuse rather than focal aneurysm morphology, normal serum IgG4, the absence of relevant mucocutaneous or ocular features, and sustained clinical and serological improvement on immunosuppression alone. The full supporting and refuting evidence for each candidate diagnosis is set out in Supplementary Table 1.

### Imaging evidence supporting active large-vessel vasculitis

Given the predominant complaint of dysphagia, upper gastrointestinal endoscopy (day 3) excluded intrinsic mucosal disease and identified extrinsic oesophageal compression; endoscopic ultrasound (day 4) localised this to a markedly dilated thoracic aorta (~ 5 cm) exerting direct mechanical pressure on the oesophageal wall. Non-contrast CT of the chest and subsequent CTA of the aorta confirmed a non-dissecting, tricuspid-valve aneurysmal dilatation of the ascending aorta (sinusal 29 mm, junctional 27 mm, tubular 47 mm) and aortic arch (45 mm), with patent arch branches and no intramural haematoma; the abdominal aorta, coeliac trunk, superior mesenteric artery, renal, and iliac arteries were of normal calibre.

**Fig. 1:**
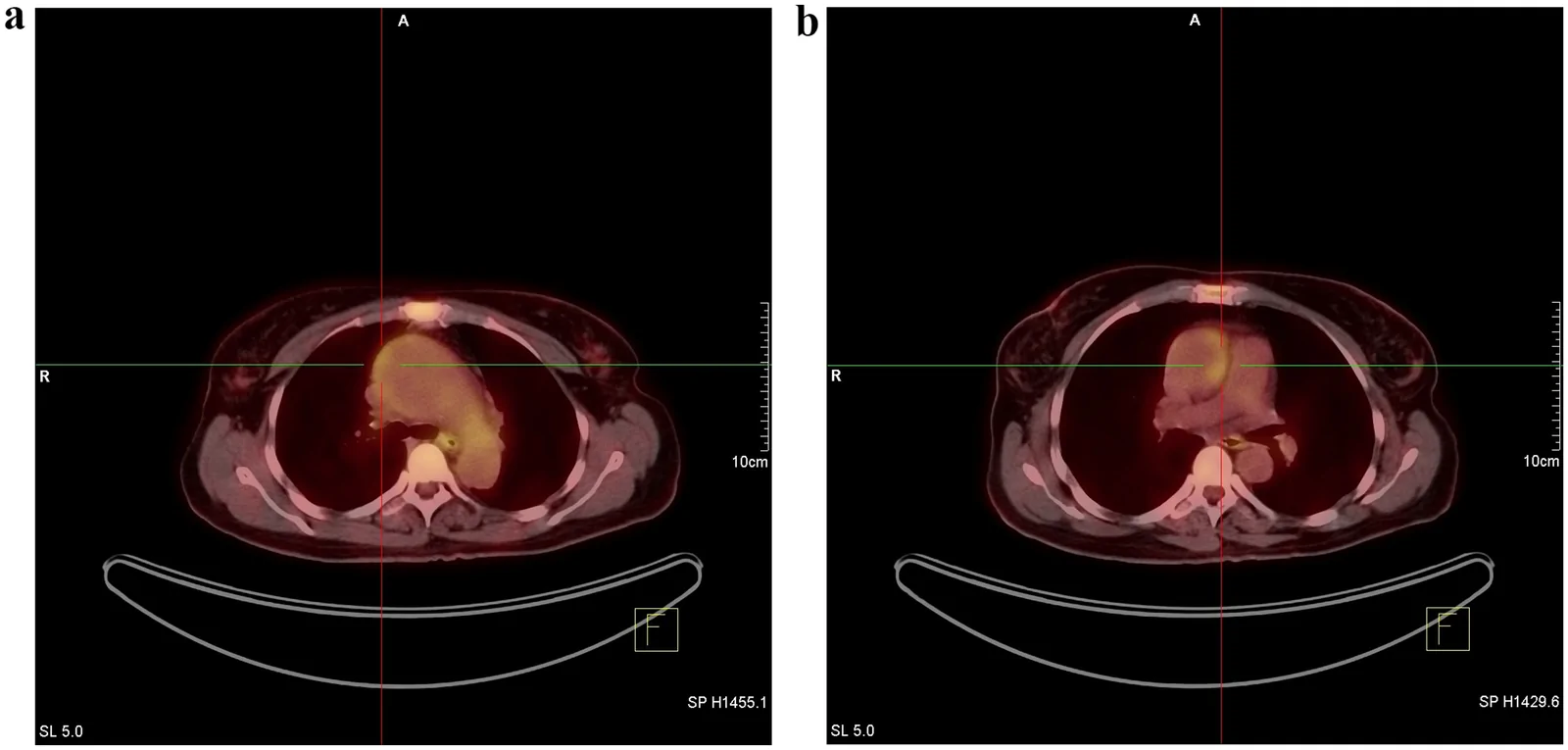
**a**, **b** Axial ¹⁸F-FDG PET/CT fusion image demonstrating FDG avidity along the lateral wall of the left ventricle, consistent with myocardial involvement in the context of active Takayasu arteritis

**Fig. 2:**
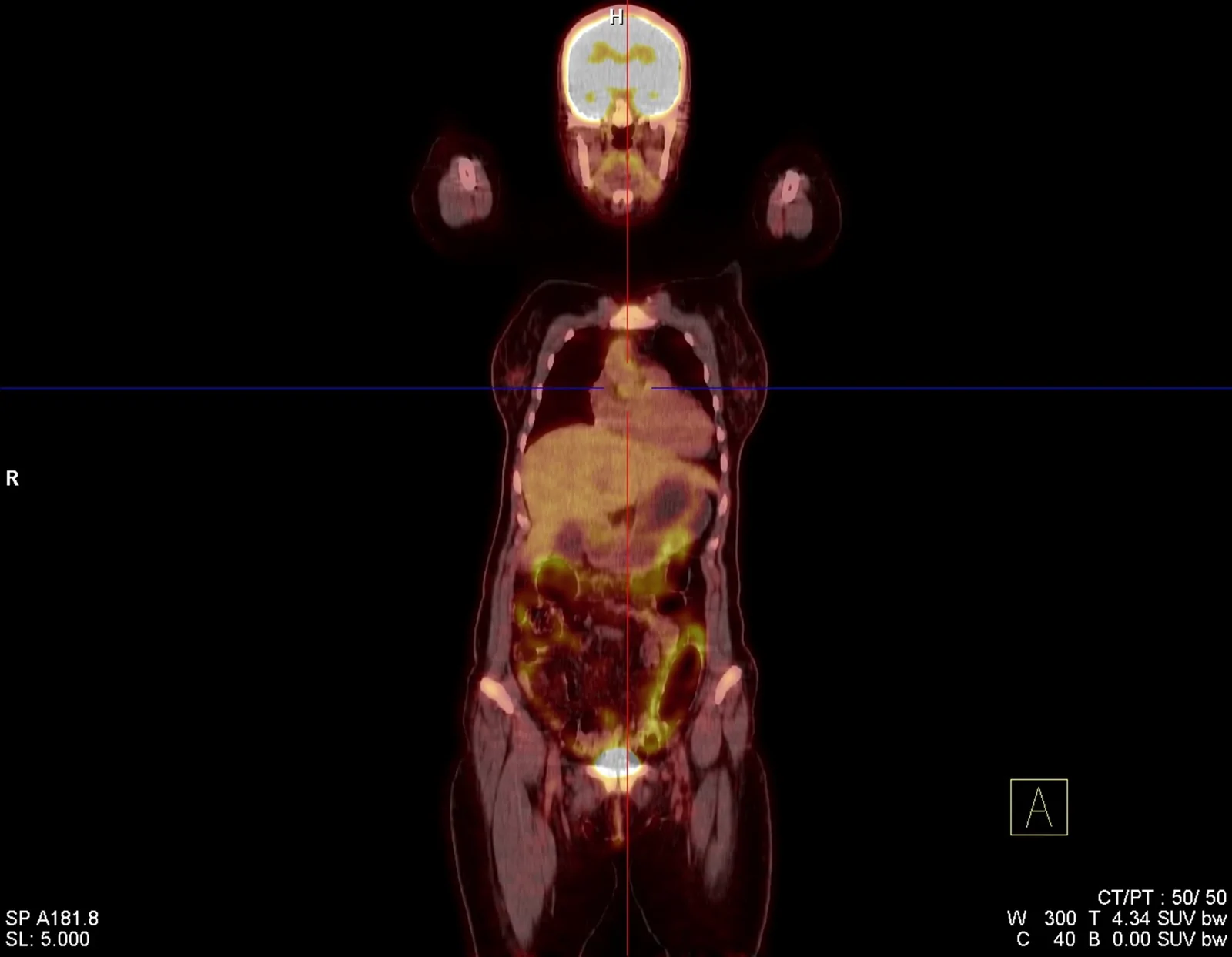
Whole-body ¹⁸F-FDG PET scan (coronal view) demonstrating pathologically increased FDG uptake along the aortic wall and its supra-aortic branches

Whole-body ¹⁸F-FDG PET, requested at this point to assess for active vascular inflammation and to exclude occult malignancy as an alternative explanation for the constitutional symptoms, showed markedly increased FDG uptake along the wall of the thoracic aorta and its supra-aortic branches, and along the left lateral wall of the left ventricle (Figs. 1a and b and 2), providing metabolic evidence of active large-vessel vasculitis rather than a purely structural or infective process.

Table 3 summarises the clinical course chronologically, integrating the key investigations, their effect on the working diagnosis, and the subsequent treatment and response.

Taken together, extrinsic oesophageal compression by a dilated, FDG-avid thoracic aorta; aortic regurgitation; elevated inflammatory markers; and systematic exclusion of infective and other inflammatory mimics (Supplementary Table 1) established a clinical diagnosis of Takayasu arteritis. Applying the item weights of the 2022 ACR/EULAR classification criteria for TA [9], the patient met the mandatory entry criterion (age ≤ 60 years with imaging evidence of large-vessel involvement) and scored for female sex and for the number of affected arterial territories on imaging (thoracic aorta and left common carotid artery), but fell below the ≥ 5-point classification threshold. As these are research classification criteria rather than diagnostic criteria for individual patients, with a reported sensitivity of 93.8% in the validation cohort [9], failure to reach the threshold did not exclude the clinical diagnosis in this patient, whose overall clinical, laboratory, and imaging picture was otherwise characteristic.

The patient’s dysphagia is therefore attributable to extrinsic oesophageal compression from Takayasu arteritis-associated aneurysmal aortic dilatation, a mechanism established only once endoscopy, endoscopic ultrasound, CTA, and FDG-PET had localised and characterised the aortic pathology and the clinical diagnosis of TA had been made.

### Treatment and clinical response

Upon confirmation of the diagnosis, prednisolone 50 mg/day and methotrexate 15 mg/week were initiated as remission-induction and steroid-sparing therapy, respectively, together with aspirin 80 mg/day and atorvastatin 40 mg/day for vascular and cardiovascular risk reduction (Table 3). At subsequent outpatient follow-up, the patient reported complete resolution of dysphagia, retrosternal chest pain, exertional dyspnoea, and musculoskeletal symptoms including morning stiffness, accompanied by a marked fall in ESR (67 to 8 mm/h) and CRP (41 to 7.1 mg/L) (Table 2). Repeat transthoracic echocardiography confirmed preserved LV function and persistent, but structurally unchanged, moderate aortic insufficiency without new valvular pathology, and serial echocardiographic surveillance was recommended.

**Table 2:** Laboratory investigations at admission, at the time of diagnosis/rheumatology referral, and at post-treatment follow-up

Parameter (reference range)	Admission	At diagnosis / referral	At follow-up
WBC, ×10³/µL (4.5–11.0)	10.14	8.90	8.00
Haemoglobin, g/dL (12.0–16.0)	10.6 ↓	10.7 ↓	11.2 ↑
MCV, fL	—	80	82
Platelets, ×10³/µL (150–400)	349	444	241
ESR, mm/h (0–20)	58 ↑	67 ↑	8 ↓↓
CRP, mg/L (< 5)	24 ↑	41 ↑	7.1 ↓↓
Creatinine, mg/dL (0.5–1.1)	0.8	—	—
ALT/AST	Normal	—	—
Urinalysis	Normal	—	—

An extended immunology and infectious screen at admission, antinuclear and anti-double-stranded DNA antibodies, complement (C3, C4, CH50), antiphospholipid antibody panel, serum angiotensin-converting enzyme, IgG and IgG4, VDRL, interferon-gamma release assay for tuberculosis, and hepatitis B/C and HIV serology, was normal or non-reactive throughout (hepatitis B surface antibody reactive, consistent with immunity), and two independent blood culture sets were negative. Total cholesterol, LDL-C, HDL-C, and triglycerides were within normal limits at admission. WBC, white blood cell count; MCV, mean corpuscular volume; ESR, erythrocyte sedimentation rate; CRP, C-reactive protein; ALT, alanine aminotransferase; AST, aspartate aminotransferase

**Table 3:** Clinical course timeline integrating the major investigations, the finding that prompted each, and the resulting diagnostic and therapeutic action, from initial symptom onset to post-treatment follow-up

Time	Investigation / event	Key finding	Effect on diagnosis / management
~T − 12 mo	Dyspnoea, chest pain, palpitations; outpatient TTE; ↑ESR/CRP	Moderate AI, LVEF 55%	IE suspected; empirical antibiotics started (outpatient)
~T − 8 to − 6 mo	New arthralgia/morning stiffness; progressive solid-food dysphagia	Systemic inflammatory and mechanical symptoms	No sustained response to antibiotics; hospitalisation arranged
Admission (Day 0)	Diastolic murmur; four-limb BP	10 mmHg inter-arm difference (non-specific)	Rheumatology referral; IE work-up continued
Day 1–4	Blood cultures ×2; immunology panel; endoscopy → EUS	Cultures/immunology negative; extrinsic oesophageal compression by ~ 5 cm dilated aorta	Bacteraemia and connective-tissue disease excluded; vascular cause of dysphagia identified
Day 5–10	CT chest → CTA aorta; initial TOE	Non-dissecting aneurysm (ascending 47 mm, arch 45 mm); AI + 6 mm cusp thickening	Aneurysmal aortopathy confirmed; IE not yet excluded, antibiotics continued
After Day 10	Whole-body ¹⁸F-FDG PET	Diffuse FDG uptake: thoracic aorta, supra-aortic branches, LV wall	Active large-vessel vasculitis confirmed metabolically
After antibiotics complete	Repeat TOE	No vegetation; AI persists structurally	Infective endocarditis excluded
Diagnosis (post-admission clinic)	Integration of all data (Supplementary Table 1); ACR/EULAR criteria applied	Mimics excluded; pattern consistent with TA	Clinical diagnosis of TA established; prednisolone 50 mg/day + methotrexate 15 mg/week + aspirin 80 mg/day + atorvastatin 40 mg/day started
Follow-up	Outpatient review	Resolution of dysphagia, chest pain, dyspnoea, MSK symptoms; ESR 67→8, CRP 41→7.1; stable moderate AI on TTE	Immunosuppression continued; serial echocardiographic surveillance planned

AI, aortic insufficiency; BP, blood pressure; CTA, computed tomography angiography; EUS, endoscopic ultrasound; IE, infective endocarditis; LV, left ventricle; LVEF, left ventricular ejection fraction; TA, Takayasu arteritis; TOE, transoesophageal echocardiography; TTE, transthoracic echocardiography

## Discussion

Takayasu arteritis (TA) is an uncommon chronic granulomatous large-vessel vasculitis with a wide range of clinical manifestations, including limb claudication, pulse abnormalities, and vascular bruits [2, 7, 15, 16]. This case is notable for its major presenting symptom, progressive solid-food dysphagia, an unusual and infrequently described initial presentation of TA. Upper gastrointestinal endoscopy excluded intrinsic mucosal disease, while endoscopic ultrasound identified extrinsic oesophageal compression by a dilated thoracic aorta (~ 5 cm), indicating a mechanical vascular cause.

This finding is anatomically consistent with ‘dysphagia aortica’, a recognised phenomenon of oesophageal compression by an ectatic or aneurysmal thoracic aorta reported predominantly in elderly patients with atherosclerotic aortopathy [13], but rarely described in the context of inflammatory large-vessel vasculitis. Aortic aneurysmal dilatation in TA, though less prevalent than stenotic or occlusive disease, is a well-documented complication with important structural and surgical implications: Sueyoshi et al. documented fusiform and saccular aortic aneurysms in a significant subset of TA patients, predominantly involving the thoracic aorta and arch [17]. Yang et al. similarly showed that aortic aneurysm in TA carries distinct prognostic and management implications compared with stenotic disease, with the thoracic aorta and arch disproportionately represented among aneurysmal phenotypes [18].

The diagnostic journey in this case illustrates how TA can closely mimic infective endocarditis, and more broadly how a structured approach to excluding infectious and other inflammatory mimics of aortitis is required before an inflammatory large-vessel process can be diagnosed with confidence (Supplementary Table 1). The combination of moderate aortic insufficiency, elevated ESR/CRP, and a focal non-coronary cusp thickening on initial TOE fulfilled several modified Duke criteria [19], justifying empirical antibiotic therapy; the diagnosis of IE was subsequently refuted by persistently negative blood cultures and the absence of vegetation on repeat TOE. ¹⁸F-FDG PET demonstrated diffuse circumferential aortic wall and branch uptake, a pattern characteristic of large-vessel vasculitis rather than focal valvular infection [6, 20], while CTA showed aneurysmal dilatation of the ascending aorta and arch without dissection; the focal valvular thickening is most parsimoniously attributed to aortic root involvement in TA rather than true vegetative endocarditis.

The 2023 EULAR recommendations for imaging in large-vessel vasculitis specify that the optimal first-line modality depends on the disease suspected: ultrasound of the temporal and axillary arteries is recommended as the first imaging test for suspected giant cell arteritis, whereas magnetic resonance imaging is recommended as the first imaging test for suspected Takayasu arteritis, with FDG-PET/CT, CT, and ultrasound as alternative modalities [21]. In this patient, CTA was obtained ahead of dedicated large-vessel MRI because the immediate diagnostic priority was to exclude infective endocarditis and characterise a mediastinal abnormality identified on CT of the chest, rather than because CTA is regarded as the default first-line investigation for suspected TA; FDG-PET/CT was added subsequently, consistent with its recognised role as an alternative modality for TA, once the clinical picture had shifted towards an inflammatory large-vessel process. PET was not essential to reaching the diagnosis, CTA had already established the anatomical diagnosis of a non-dissecting aneurysm, and the clinical and serological picture had already favoured an inflammatory over an infective process, but it contributed two things that anatomical imaging and serology alone could not: direct metabolic evidence that the aortic wall was the site of ongoing active inflammation rather than a quiescent structural lesion, supporting the decision to proceed to immunosuppression; and delineation of extra-aortic and myocardial involvement not suspected on clinical grounds. Given its cost and limited availability in many settings, FDG-PET/CT is likely to be most useful when diagnostic uncertainty persists between an inflammatory and an infective or purely structural process after first-line clinical assessment and vascular imaging, rather than as a routine investigation in every suspected case of large-vessel vasculitis.

A median delay from symptom onset to diagnosis ranging from one to several years has been reported across international TA cohorts [15, 22], reflecting the disease’s insidious onset and non-specific early manifestations, as illustrated here by the approximately one-year symptom evolution and the initial misattribution to infective endocarditis. On formal application of the 2022 ACR/EULAR classification criteria for TA [9], our patient scored for female sex and for the number of affected arterial territories on imaging but did not reach the ≥ 5-point classification threshold, lacking limb claudication, an arterial bruit, a reduced peripheral or carotid pulse, an inter-arm blood pressure difference ≥ 20 mmHg, paired-artery involvement, and abdominal aorta/renal/mesenteric disease. We highlight this because the ACR/EULAR instrument is a research classification tool with reported sensitivity of 93.8% in its validation cohort [9], not a diagnostic test, and failure to meet the threshold does not exclude the clinical diagnosis of TA in a patient whose overall clinical, laboratory, and imaging picture is otherwise characteristic, as here [6, 20]. The arch- and ascending aorta-predominant aneurysmal phenotype observed is concordant with the female sex-associated vascular distribution pattern documented across multiple international TA series [3–5].

Thoracic aortic aneurysm, as in the present case, is a recognised but comparatively uncommon structural complication of TA. Table 4 summarises representative published case reports, case series, and the largest dedicated surgical cohort to date, illustrating the range of presentations, treatment approaches, and outcomes reported; this is intended as an illustrative summary of the literature rather than an exhaustive systematic review.

**Table 4:** Representative published reports of thoracic aortic aneurysm in Takayasu arteritis

Source (year)	Study type / *n*	Aneurysm location	Treatment and outcome
Sueyoshi et al. (2000) [17]	Retrospective CT cohort of TA patients	Fusiform and saccular aneurysms, predominantly thoracic aorta and arch	Aneurysms could enlarge over time, including during clinical remission; underscores need for serial imaging surveillance
Yang et al. (2017) [18]	Retrospective cohort of TA patients with aortic aneurysm	Thoracic aorta and arch over-represented among aneurysmal phenotypes	Aneurysmal phenotype carries distinct prognostic/management implications vs. stenotic/occlusive TA
Robinson et al. (2005) [23]	Case report + literature review	Rapidly progressive (‘fulminant’) thoracic aortic dilatation (‘mega-aorta’)	Combined immunosuppressive and staged surgical management; illustrates risk of rapid progression in active disease
Simkhada et al. (2023) [24]	Case report	Aortic root/ascending aorta aneurysm with severe AR	Modified Bentall procedure; diagnosis confirmed on surgical histopathology; favourable short-term outcome
Fan et al. (2017) [25]	Case report	Recurrent ascending aortic aneurysm after prior double valve replacement	Successful redo aortic surgery; highlights need for long-term surveillance after repair
Obitsu et al. (2010) [26]	Case report	Extensive thoracic aneurysm (arch to descending aorta)	Staged hybrid repair (arch replacement + TEVAR); favourable outcome to ~ 5-year follow-up
Ergi et al. (2025) [27]	Retrospective surgical cohort, *n* = 31 (1994–2022)	Ascending aortic aneurysm most common presentation (71%)	Ascending aorta ± aortic valve replacement; early mortality 6.5%; 10-year survival ~ 87%
Present case	Case report	Ascending aorta (47 mm) and arch (45 mm) aneurysm, non-dissecting, with moderate AR and dysphagia	Medical therapy alone (glucocorticoid + methotrexate); rapid clinical/serological remission; surveillance planned

AR, aortic regurgitation; TEVAR, thoracic endovascular aortic repair

The patient’s rapid and sustained clinical and serological response to glucocorticoid- and methotrexate-based immunosuppression (Case Presentation; Tables 2 and 3) is concordant with the established evidence base for remission induction in TA, in which high-dose glucocorticoids (0.5–1 mg/kg/day) remain the cornerstone of initial therapy despite substantial relapse rates on tapering [6, 7, 15, 20]. Persistent moderate aortic insufficiency on follow-up echocardiography reflects established structural vascular damage rather than ongoing inflammation, a distinction of fundamental importance in monitoring TA; as Sueyoshi et al. demonstrated, aortic aneurysms in TA may progress even during clinical remission [17], and the surgical outcome data summarised in Table 4 [23–27] underscore the need for long-term serial imaging surveillance with individualised thresholds for intervention.

## Conclusion

In conclusion, this case demonstrates that TA should be considered in middle-aged women with unexplained dysphagia, aortic regurgitation, elevated inflammatory markers, and mediastinal aortic enlargement, even when the initial working diagnosis is infective endocarditis, and even when formal classification-criteria thresholds are not met. Definitive diagnosis required systematic integration of clinical assessment, including four-limb blood pressure measurement, with multimodality imaging comprising endoscopy, EUS, CT angiography, TOE, and ¹⁸F-FDG PET, together with a structured exclusion of infectious and other inflammatory mimics of aortitis. Early recognition and immunosuppressive therapy can achieve rapid clinical remission, though structural vascular complications necessitate ongoing surveillance.

## Supplementary Information

Below is the link to the electronic supplementary material.

Supplementary Material 1

## Data Availability

No datasets were generated or analysed during the current study.

## Abbreviations

ACE: Angiotensin-converting enzyme
ACR/EULAR: American College of Rheumatology/European Alliance of Associations for Rheumatology
AI: Aortic insufficiency
ALT: Alanine aminotransferase
AMVL: Anterior mitral valve leaflet
anti-dsDNA: Anti-double-stranded DNA antibodies
APS: Antiphospholipid antibody
AR: Aortic regurgitation
AST: Aspartate aminotransferase
BP: Blood pressure
C3/C4: Complement components 3 and 4
cDMARD: Conventional disease-modifying antirheumatic drug
CH50: Total haemolytic complement activity
CRP: C-reactive protein
CT: Computed tomography
CTA: Computed tomography angiography
ESR: Erythrocyte sedimentation rate
EUS: Endoscopic ultrasound
FDG: Fluorodeoxyglucose
IE: Infective endocarditis
IgG: Immunoglobulin G
IMH: Intramural haematoma
LV: Left ventricular
LVEF: Left ventricular ejection fraction
LVH: Left ventricular hypertrophy
MCV: Mean corpuscular volume
MR: Mitral regurgitation
PAP: Pulmonary artery pressure
PET: Positron emission tomography
PR: Pulse rate
RR: Respiratory rate
RV: Right ventricular
SMA: Superior mesenteric artery
SpO₂: Oxygen saturation
TA: Takayasu arteritis
TEVAR: Thoracic endovascular aortic repair
TOE: Transoesophageal echocardiography
TR: Tricuspid regurgitation
TTE: Transthoracic echocardiography
VDRL: Venereal Disease Research Laboratory
WBC: White blood cell
