# Supplementary material for: Takayasu arteritis presenting with dysphagia and morning stiffness: a diagnostic challenge in a middle-aged woman
Source: Egypt Heart J. 2026 Sep 5;78:63. doi: 10.1186/s43044-026-00774-z (PMC13546264; doi:10.1186/s43044-026-00774-z)
Supplement: Supplementary file 1 [file 43044_2026_774_MOESM1_ESM.docx]

| **Candidate diagnosis** | **Features supporting** | **Features against** | **Assessment** |
| --- | --- | --- | --- |
| Infective endocarditis | ↑ESR/CRP; aortic regurgitation; 6 mm non-coronary cusp thickening on initial TOE | Two negative blood culture sets; no vegetation on repeat TOE after antibiotic course; no predisposing valve lesion, IV drug use, or embolic phenomena; no fever throughout course | Excluded |
| Infectious aortitis / infected aortic aneurysm (incl. culture-negative organisms, e.g. Coxiella, Bartonella) | Aneurysmal dilation; systemic inflammation; prior antibiotic exposure could partially treat/mask infection | No fever; no risk factors (IVDU, recent instrumentation, immunosuppression, prosthetic material); morphology diffuse and fusiform rather than the focal/saccular pattern typical of mycotic aneurysm; sustained clinical and serological improvement on immunosuppression alone, without antimicrobials | Considered unlikely |
| Giant cell arteritis (large-vessel pattern) | Constitutional symptoms; ↑inflammatory markers; large-vessel FDG uptake | Age 45 years, below the ≥50-year absolute age requirement of the 2022 ACR/EULAR GCA criteria; no cranial features (headache, scalp tenderness, jaw claudication, visual symptoms) | Excluded, primarily on age and absence of cranial features |
| IgG4-related aortitis/periaortitis | Aortic disease is a recognised IgG4-related manifestation | Normal serum IgG4; no other organ involvement (pancreas, biliary tree, retroperitoneum, salivary/lacrimal glands); typical IgG4-related periaortitis more often affects the infrarenal abdominal aorta | Considered unlikely; formal exclusion would require tissue biopsy, which was not performed/available |
| Behçet disease | Can cause large-vessel vasculitis | No oral or genital ulcers, no ocular involvement, no relevant skin lesions, pathergy not tested; does not meet International Criteria for Behçet's Disease | Excluded clinically |
| Isolated / idiopathic aortitis | Recognised entity when aortitis is found without an identifiable systemic association, typically on surgical histopathology | Additional systemic features present (inflammatory arthralgia/morning stiffness) and extra-aortic (carotid) FDG uptake, favouring a systemic vasculitic process; no surgical specimen available for histopathology in this medically managed patient | Less likely than a systemic large-vessel vasculitis, but cannot be entirely excluded without histopathology |
| Degenerative/atherosclerotic aneurysm ('dysphagia aortica') | Recognised cause of extrinsic oesophageal compression, typically in older patients | Relatively young age (45); no significant atherosclerotic risk factors beyond mild hypertension; ↑inflammatory markers; diffusely FDG-avid aortic wall | Unlikely as the primary driver, though degenerative change may coexist |

*Supplementary Table 1. Structured differential diagnosis with supporting and refuting evidence for each candidate diagnosis.*
